# P-1855. Dissecting the contribution of Herpesviruses in the emergence of depressive symptoms in Alzheimer's Disease through multi-omics data

**DOI:** 10.1093/ofid/ofae631.2016

**Published:** 2025-01-29

**Authors:** Anna Onisiforou, Panos Zanos

**Affiliations:** University of Cyprus, Nicosia, Nicosia, Cyprus; University of Cyprus, Nicosia, Nicosia, Cyprus

## Abstract

**Background:**

The association between members of the Herpesviridae family and the development of Alzheimer's Disease (AD) and Depression is well-documented. However, the specific pathogenic mechanisms through which herpesviruses contribute to the co-occurrence of AD and major depression remain poorly elucidated.

**Methods:**

To explore this hypothesis, we employed an integrated multi-omics bioinformatics approach combining virus-host protein-protein interactions (PPIs) and transcriptomic data. This method was utilized to dissect the complex interplay between herpesvirus infections and the host biological pathways that might be jointly involved in AD and major depression.

**Results:**

Our findings indicate significant overlaps in the herpesvirus-modulated pathways in AD and those implicated in major depression. Notably, specific herpesviruses were found to influence pathways related to neuroinflammation, synaptic function, and mood regulation, which are critical in both AD and depression. Additionally, co-infection with multiple Herpesviridae members was associated with increased susceptibility to developing comorbid AD and major depression.

**Conclusion:**

This study supports the hypothesis that herpesviruses contribute to the pathogenesis of both AD and major depression through shared biological pathways. These insights highlight the potential of targeting these pathways in therapeutic strategies for patients suffering from both conditions. Furthermore, our results underscore the importance of considering viral infections in the clinical management of AD and comorbid depressive symptoms.

**Disclosures:**

All Authors: No reported disclosures

